# Supplementary material for: Curated and harmonised transcriptomics datasets of interstitial lung diseases
Source: Data Brief. 2025 Oct 14;63:112139. doi: 10.1016/j.dib.2025.112139 (PMC12581653; doi:10.1016/j.dib.2025.112139)
Supplement: Supplementary file 1 [file mmc1.zip › Supplementary_material/RNA-seq/fastQC reports/GSE124685/TRIMMED_SRR8397186_fastqc.html]

TRIMMED\_SRR8397186.fastq.gz FastQC Report 

FastQC Report

Sat 3 Dec 2022  
TRIMMED\_SRR8397186.fastq.gz

## Summary

- Basic Statistics
- Per base sequence quality
- Per sequence quality scores
- Per base sequence content
- Per sequence GC content
- Per base N content
- Sequence Length Distribution
- Sequence Duplication Levels
- Overrepresented sequences
- Adapter Content

## Basic Statistics

| Measure | Value |
| --- | --- |
| Filename | TRIMMED\_SRR8397186.fastq.gz |
| File type | Conventional base calls |
| Encoding | Sanger / Illumina 1.9 |
| Total Sequences | 3248826 |
| Sequences flagged as poor quality | 0 |
| Sequence length | 35-356 |
| %GC | 51 |

## Per base sequence quality

## Per sequence quality scores

## Per base sequence content

## Per sequence GC content

## Per base N content

## Sequence Length Distribution

## Sequence Duplication Levels

## Overrepresented sequences

| Sequence | Count | Percentage | Possible Source |
| --- | --- | --- | --- |
| GGGCCACGAGCTGAGTGCGTCCTGTCACTCCACTCCCATGTCCCTTGGGA | 70324 | 2.1645973037644985 | No Hit |
| AAGCTCAGGGAGAGCCCTGTTAGGGCCGCCTCTGGCCCTAGTCTCAGACC | 57809 | 1.7793812287884916 | No Hit |
| GGGAATGTCTACGTGCGTATGCACGTGGCACTCTCTGCC | 25886 | 0.7967801291912833 | No Hit |
| GCTGCTACAGGAGAATAGCAGACAGGTATAGTTAAGAAACAACTTTATTT | 20889 | 0.6429707223470879 | No Hit |
| ACACTGAGGACTCTGTTCCTCCCCTTTCCGCCTAGGGGAAAGTCCCCGGA | 20282 | 0.624287050152886 | No Hit |
| CTAAGTGCTGTGTTGTCGTTCCCCCTGCTTAAAATAAAGTTGTTTCTTAA | 20268 | 0.6238561252587858 | No Hit |
| ACTTGATCCCAACTCATCTCTCATTTATTTCGGCTTCTTTTATTCCAGGA | 12966 | 0.39909801263594913 | No Hit |
| TCTCTGGGCTTCTATTTCGACCGCGATGATGTGGCTCTGGAAGGCGTGAG | 11341 | 0.3490799445707465 | No Hit |
| AAAGCCAAGAAAACCAACGATGCCAGATACTGAATTTCTATAGCACTGAC | 10938 | 0.3366754636905762 | No Hit |
| TTTACACCTGCTAGGGTGTTCAAAGGTCAGTGCTATAGAAATTCAGTATC | 10151 | 0.31245132857222885 | No Hit |
| AATAAAAGCGAAAAGAAATGAAAATGTTACACTACATTAATCCTGGAATA | 8851 | 0.2724368741200668 | No Hit |
| AGCGGTTCCGCTGCCCTGAGGCACTCTTCCAGCCTTCCTTCCTGGGCATG | 7769 | 0.23913253587603645 | No Hit |
| AAAAGTGATGGACTGCCCATTGCTGGAGAAGACCTTCTCTCCTACTGTCA | 7582 | 0.2333766105048408 | No Hit |
| GGGAATGTCTACGTGCGTATGCACGTGGCACTCTCT | 6834 | 0.21035290902005835 | No Hit |
| GGTTATAATCATTGGCAATGTTGTAATCAACAACCCATGTTATTTCTACA | 6465 | 0.19899496002556 | No Hit |
| TGGTTCCTGGCAAGCCCATGTGTGTTGAGAGCTTCTCAGACTATCCACCT | 6393 | 0.1967787748559018 | No Hit |
| TCACACTTCATGATGGAGTTGAAGGTAGTTTCGTGGATGCCACAGGACTC | 6313 | 0.1943163468896149 | No Hit |
| ATTCTTCCTACCCATGAGCATGGAATGTTCTTCCATTTGTTTGTATCCTC | 6099 | 0.18772935207979743 | No Hit |
| ACACCCACCGCAACTGTCTGTCTCATATCACGAACAGCAAAGCGACCCAA | 5973 | 0.18385102803289557 | No Hit |
| ATCAAATCCTGCAGACAAGGGGAGCCCTCAGTCTGCAGGGCTCCATAATG | 5701 | 0.17547877294752012 | No Hit |
| TGACTGGTGATGCTGATATTGGAGTGCATCTGAGGCTGTTGTAGAAATAA | 5698 | 0.17538643189878436 | No Hit |
| CGCTGCACTGACCTTCTTCCAAGCCTCAGTTCCTGTTCTAGGAACTTGAG | 5556 | 0.1710156222586251 | No Hit |
| ACACTGAGGACTCTGTTCCTCCCCTTTCCGCCTAGGGAAAGTCCCCGGAC | 5435 | 0.16729119995961617 | No Hit |
| ACGCTCGTAGCCCTCGCGCTTCTCCTCGGCCAGTTCGCGGAAGAAGTGGC | 5406 | 0.16639856982183718 | No Hit |
| ACGCTCGTAGCCCTCGCGCTTCTCCTCGGCCAATTCGCGGAAGAAGTGGC | 5292 | 0.16288960996987834 | No Hit |
| TGAATCTTGAACTGAGTTCCACTTGTAAACTTCTTGTTTCTTGTGGTTCC | 5286 | 0.16270492787240684 | No Hit |
| CCACCTTCTCCATCGGCTCCACTGGCCTCGTGGTGTATGACTACCAGCAG | 5259 | 0.161873858433785 | No Hit |
| TCTTTCTGGCCTGGAGGCTATCCAGCGTACTCCAAAGATTCAGGTTTACT | 5195 | 0.1599039160607555 | No Hit |
| ATGATGTAGCAGCAGGTGCCAGGGGCTGGCTTGTAGGCGATCAGCAGCTG | 5010 | 0.15420955138871703 | No Hit |
| AAGCTCAGGGAGAGCCTGTTAGGGCCGCCTCTGGCCCTAGTCTCAGACCT | 4814 | 0.14817660287131412 | No Hit |
| TCTGTCTTCTTCAGTTTCGACTTATCGAATTTCTCGATCTCAGCCATATC | 4594 | 0.14140492596402515 | No Hit |
| TGTCTGTCTGTGTTTTGCATTCAGATCTTGCTATCCACACAAACATCATG | 4235 | 0.1303547804653127 | No Hit |
| ACACTGAGGACTCTGTTCCTCCCCTTTCCGCCTAGGGAAAGGTACCCGGC | 4131 | 0.12715362410913972 | No Hit |
| ACATAGCAATTCAGGAAATTTGACTTTCCATTCTCTGCTGGATGACGTGA | 4050 | 0.12466041579327425 | No Hit |
| AAGACCAACGTCAAGGCCGCCTGGGGTAAGGTCGGCGCGCACGCTGGCGA | 3925 | 0.12081287209595097 | No Hit |
| GGGCCACGAGCTGAGTGCGTCCTGTCACTCCACTCCCATGTCCCTTGGGG | 3896 | 0.11992024195817197 | No Hit |
| CTTCCGCATAGCTGCTGTGGTCAAAAAGGAGCCCAGAGTGACAGTTTTCC | 3820 | 0.11758093539019941 | No Hit |
| ACAAGCAGTTCACAGTAGAACACTGCAGACTGCAGGGCATTTTCTGGCTA | 3793 | 0.11674986595157759 | No Hit |
| AGTGTGGCGATTCCTCAGGGATCTAGAACTAGAAATACCATTTGACCCAG | 3757 | 0.1156417733667485 | No Hit |
| TCAGAAAGGTCACGTCCTGCTCTTGGTCCTTGTCAGTGCCATGTTCTGTG | 3702 | 0.11394885413992624 | No Hit |
| TCTTCAAGGAGAACTACAAACCACTGCTCAAGGAAATAAAAGAGGATACA | 3515 | 0.10819292876873061 | No Hit |
| ACAAGCAGTTCACAGTAGAACACTGCAGACTGCAGGGCATTTTCAGGCTA | 3502 | 0.10779278422420899 | No Hit |
| TACTCGTGCGCCTCGCTTCGCTTTTCCTCCGCAACCATGTCTGACAAACC | 3452 | 0.10625376674527967 | No Hit |
| GGCCTTCTTCTGGCGCAGAGGAAGCAGGCGCATCTGGGTGTGGGCAATGA | 3443 | 0.10597674359907241 | No Hit |
| TACATCAAAGATTACATGAAATCAATCAAAGGGAAACTTGAAGAACAGAG | 3352 | 0.10317573178742105 | No Hit |
| TCTCTGGCTTCTATTTCGACCGCGATGATGTGGCTCTGGAAGGCGTGAGC | 3327 | 0.10240622304795641 | No Hit |

## Adapter Content

Produced by FastQC (version 0.11.7)
